# Quantifying the effects of the break up of Pangaea on global terrestrial diversification with neutral theory

**DOI:** 10.1098/rstb.2015.0221

**Published:** 2016-04-05

**Authors:** Sean M. R. Jordan, Timothy G. Barraclough, James Rosindell

**Affiliations:** 1 Department of Life Sciences, Imperial College London, Silwood Park Campus, Buckhurst Road, Ascot SL5 7PY, UK; 2 Centre for Biodiversity and Environment Research, Department of Genetics, Evolution and Environment, University College London, Gower Street, London WC1E 6BT, UK

**Keywords:** fossil record, coalescence, biodiversity, speciation, dispersal, continental drift

## Abstract

The historic richness of most taxonomic groups increases substantially over geological time. Explanations for this fall broadly into two categories: bias in the fossil record and elevated net rates of diversification in recent periods. For example, the break up of Pangaea and isolation between continents might have increased net diversification rates. In this study, we investigate the effect on terrestrial diversification rates of the increased isolation between land masses brought about by continental drift. We use ecological neutral theory as a means to study geologically complex scenarios tractably. Our models show the effects of simulated geological events that affect all species equally, without the added complexity of further ecological processes. We find that continental drift leads to an increase in diversity only where isolation between continents leads to additional speciation through vicariance, and where higher taxa with very low global diversity are considered. We conclude that continental drift by itself is not sufficient to account for the increase in terrestrial species richness observed in the fossil record.

## 1. Introduction

**Figure 1.**
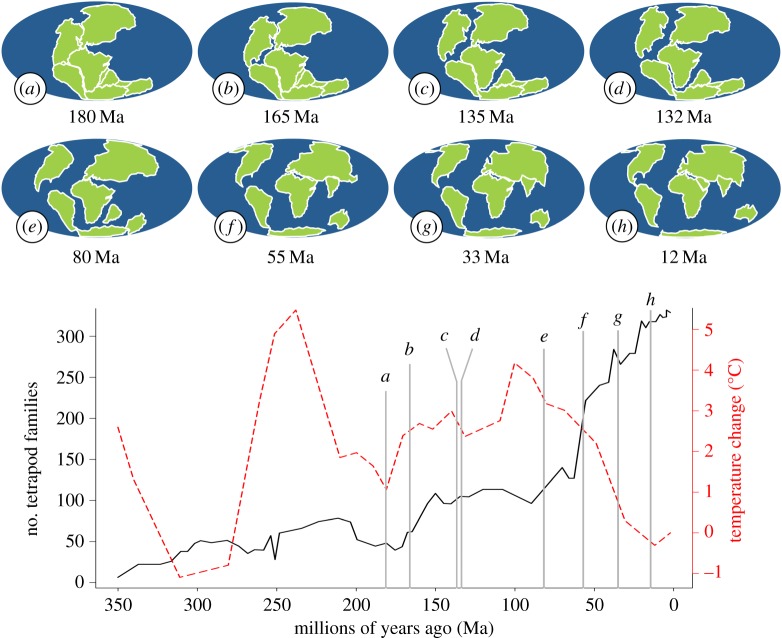
Phanerozoic tetrapod diversity [1] and change in global average temperature [2]. Above are approximate depictions of land mass positions at (*a*) 180, (*b*) 165, (*c*) 135, (*d*) 132, (*e*) 80, (*f*) 55, (*g*) 33 and (*h*) 12 Ma. Based on information in Dietz & Holden, Macdonald *et al*. and Veevers [3–5].

Numbers of taxa in the fossil record show a dramatic increase through geological time. For example, terrestrial tetrapods are well documented in the fossil record and show a sharp increase in diversity over time (figure 1). Periods of particularly steep increase in diversity coincide with events such as the initial break up of Pangaea at *ca* 180 million years ago (Ma) [3]. Other taxa also proliferated over similar timescales. These include, for example, the seed-bearing plants which underwent a massive increase in diversity [6–8]. Similarly, beetles (Coleoptera) experienced a significant rise in species richness around the early Cretaceous [8], possibly associated with the evolution of the first angiosperms [9].

One explanation for this apparent increase in diversity is bias in the fossil record. Older rock formations and specimens have had a longer time to deteriorate [10]. Furthermore, earlier period substrates might be less well suited for the preservation of organic material [11]. For example, the notable increase in diversity during the Phanerozoic has been attributed to a sampling bias caused by the availability of outcropping sedimentary rock [12]; this observation remains robust despite continued re-classification of specimens' taxa, origination and extinction dates [13,14]. The trend in diversity of Coleoptera over time may similarly reflect specimen preservation in amber [15]. A further sampling bias may arise because it is easier to classify taxa with present-day representatives than those with no living relatives, leading to elevated numbers of described taxa nearer the present day. Together, these sampling effects may lead to significantly elevated estimates of diversity towards the present day.

Alternatively, trends in the fossil record could reflect true changes in diversity. Diversity might have increased, because the system has not yet reached equilibrium [16] or because conditions have changed over time and led to increased net diversification rates. One explanation would be if ‘diversity begets diversity’. Ongoing coevolution among different organisms could lead to increasingly specialized ecological roles and niche filling over time. Perhaps the most dramatic and obvious change in the theatre for terrestrial diversification of the already present communities during the Phanerozoic was the change in the distribution of continents. The early expansion of terrestrial tetrapods occurred in the context of a single global continent, which had split into seven major isolated continents by the end of the Cretaceous (figure 1). Continental drift has a strong influence on clade geographical distributions [17], and has led to opportunities for vicariance and thereby potentially increased global speciation rates and species richness. Analogous to the effects of range fragmentation on the isolation and divergence of individual populations [18], could the breakup of Pangaea explain temporal trends in the richness of terrestrial organisms as a whole?

Here we investigated the effects of continental drift on species richness using ecological neutral theory [19], which assumes that the properties of an individual are independent of its species identity. Use of neutral theory in this context highlights the effects of dispersal, speciation and continental drift on their own without any contribution from other possible explanations for the observed patterns (e.g. niche generation). Neutral theory is typically only applied to guilds of species from the same trophic level (e.g. tropical moist forest trees). A system composed of many different non-interacting guilds (or niches) does, however, usually make the same predictions [20] thus justifying the use of neutral theory, at least as an indicator for baseline behaviour. A further advantage of using neutral theory is that techniques exist based on coalescence to make it tractable for large-scale simulations of spatially complex situations [21]. Neutral theory has previously been used over geological timescales to study the effects of sea-level fluctuations on the available habitable zone of shallow-marine communities over a 2.5 million year (Myr) timescale [22]. In contrast, our present focus on terrestrial vertebrates is different and runs over a much longer timescale [23] of approximately 180 Myr [24]. We first study a simple neutral model with no dispersal between continents from which some results can be analytically derived. We relate these findings to those predicted by elementary species area relationship (SAR) methods that give species richness as a function of habitat area according to a power law. We also perform neutral simulations that incorporate a scaled down, but otherwise realistic, model of the movement, splitting and merging of land masses over the last 180 Myr in a spatially explicit manner with dispersal. We compare these geologically accurate models with simpler ones, and consider the effects of different approaches to speciation that formally interact with intercontinental dispersal. In particular, we compared a model in which speciation is independent of dispersal with a model where dispersal between continents triggers additional speciation through vicariance. By incorporating neutral ecological principles over geological timescales, this work takes another step towards bridging the gap between ecology and historical biogeography [25].

## 2. Methods

Ecological neutral theory [19] forms the basis for our model. Neutral models make the assumption that an individual's behaviour is independent of its species identity. The best-known model in this class proceeds in discrete time steps from an arbitrary initial condition. In each time step, an individual within the community dies and is replaced by the offspring of another remaining in the community; this results in ‘zero-sum’ dynamics and a constant number of individuals given by *J*_M_ the ‘metacommunity size’. Each individual has an equal probability of reproducing to fill the gap left after a death, so species with a greater number of individuals are more likely to be chosen: individuals are equivalent but species may be different in so far as they may be represented by different numbers of individuals. To prevent diversity from declining over time to a monoculture, any new-born individual founds a new species with probability *v* (speciation). This ‘point mutation’ speciation model is phenomenological given that a protracted and geographically extended process occurs in reality [25], but it is the simplest and most often used speciation method in neutral models. In this study, we will also incorporate a vicariance component to speciation in a more advanced model.

### (a) Analytical approximations of the basic neutral model

The number of species *S* in a sample of size *J* taken from the simplest (non-spatial) neutral model described above is closely approximated [19] by the formula
2.1
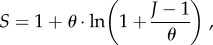
where *θ* is the fundamental biodiversity number, which is given by
2.2
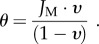


Note that the additional factor of 2 sometimes included in the definition of *θ* only applies in the case of non-overlapping generations such as annual plants [26]. Now, take *x* to indicate the probability of sampling an individual from the system so 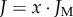 and combine with equations (2.1) and (2.2) to give
2.3
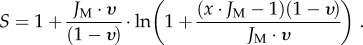
Provided that speciation is rare (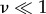 is small so that 1 − *v* ≈ 1) and the number of individuals sampled is not small (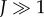 so that 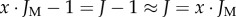), both of which are true in most realistic scenarios, equation (2.3) can be approximated as
2.4
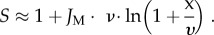
We will use equation (2.4) to establish an upper bound to the total gain in species richness that may result from the break up of Pangea into smaller continents of the same total area. To do so, we compare the total diversity in *N* independent land masses with no overlapping species to that of a single supercontinent containing the same total number of individuals. The more realistic case where new continents are partially connected and arose from a common origin, so that some species may exist in multiple continents, can only decrease the potential for such a gain in species richness. The upper bound to total gain in species richness can be shown (see electronic supplementary material, appendix 1) to be approximated by *N* − 1, irrespective of *x* and *v*. This number will only appear significant when the total diversity is small compared with the number of continents. We varied the speciation rate, *v*, to determine the effects on species richness (the probability of sampling, *x*, did not have a qualitative effect). Extremely low values of *v* yield low values of total diversity. These values are not realistic for global species richness but could reflect possible origination rates of higher taxa such as families or orders, which are fewer in number and may thus be impacted significantly by an increase of *N* − 1.

### (b) Connection to species area relationships

As an alternative to neutral theory, empirical SARs give approximate diversity as a function of area. SARs are typically described by the power-law relationship *cA^z^*, which gives a straight line in logarithmic space characterized by the parameters *c* and *z* [27]. The parameter *z* is typically 0.2 at intermediate scales. At sufficiently large scales where species range sizes are negligible compared with the size of the area *A*, almost everything will be endemic and doubling the area will double the diversity giving *z* = 1 [19,28–30]. Now consider Pangaea with area *A* and diversity *S* splitting into *N* smaller land masses with the same total area. Take *p_i_* for 1 ≤ *i* ≤ *N* to indicate the proportion of Pangaea's area left in each smaller land mass; so the areas of the smaller land masses are given by *p_i_.A* and 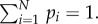 The total species richness *S*_tot_ across all *N* fragments assuming no overlap of species is given by
2.5
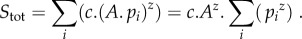


Using the formula for the original richness of Pangaea *S* = *cA^z^* provides us with a simple way to calculate the ratio of richness after the break up of Pangaea to the original richness based on *z* and the proportional size
2.6
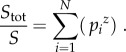
We can see that when *z* = 1 there is no change in species richness as a result of the break up of Pangaea in broad agreement with equation (2.4). At the scale of present-day continents, however, values of *z* typically do not quite reach unity. Values of 0.8 are more typical for birds, and yet lower values are seen for mammals and amphibians [28]. For *z* < 1, there is a clear gain in diversity given by equation (2.6); we will calculate this explicitly for *z* = 0.8, 0.2 and compare it with empirical values from figure 1.

### (c) Basic simulation methods

The ‘size’ of a community in our model is measured in terms of the number of individual organisms occupying it. In order to root this in reality, we require a density, *δ*, of individuals per square kilometre. Similarly, the passage of time is measured in terms of the number of individual births and deaths that have taken place; this may be related to actual time using the generation length in years of an individual, *g*. We would expect there to be *J*_M_/2*g* individual births per year, and an equal number of deaths, the division by 2 here is to account for overlapping generations.

We conducted initial numerical evaluations in which one continent is simulated to equilibrium based on the model rules, then split into many continents each of which is isolated from the rest and simulated independently. The simulations capture both the change in diversity through time and the natural stochasticity in species richness (the analytical formulae described above only give the mean result and are not time-dependent). Pangaea was initialized at its natural equilibrium species richness assuming no further movement of land masses prior to the formation of Pangaea.

We simulated our model using a coalescent approach [21] to increase efficiency. Compared with straightforward application of the model rules, this approach is typically orders of magnitude faster [21]. Despite the considerable advantages of coalescence, it is still only feasible to study scaled down versions of reality where *g* may be longer and *δ* smaller than in reality. We explore a range of parameter values and show consistent results throughout. Based on this, coupled with previous discoveries of scaling results in neutral theory [19,30–32], we do not expect scaling down to qualitatively affect our findings. All simulated data points were repeated 20 times using high-performance computing.

### (d) Incorporating dispersal and connectivity

In order to expand our neutral model to a spatial environment consisting of multiple continents connected by dispersal, we must define the probability that a habitat gap will be filled by the offspring of an individual in another land mass. We assume each continent (or community) is homogeneously mixed, whereas the system as a whole may contain spatial structure depending on dispersal. We use a ‘colonization matrix’ (*C_i_*_,*j*_(*i*, *j* ≤ *N*)) to describe the probability that a dead individual in community *i* will be replaced with the offspring of an individual in community *j* within a system containing a total of *N* communities. In the case where *i* is not equal to *j*, we define *C_i,j_* as
2.7
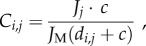
where the variable *c* controls the overall connectivity of the system, *J_j_* represents the number of individuals in the *j*th community, and *d_i_*_,*j*_ gives the distance (km) between the *i*th and *j*th communities involved. Equation (2.7) can be understood intuitively as follows:

— after each death, individuals may either recruit offspring within the same landmass (*i* *=* *j*), or in one of the others (*i* ≠ *j*);
— in the case where (*i* ≠ *j*) and dispersal occurs between continents, the probability of a colonization from continent *j* to continent *i* will be proportional to the number of individuals *J_j_* in continent *j* as a fraction of the total number of individuals in the system: 
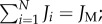
— the probability of successfully colonizing and replacing a dead individual decreases as distance increases between the two concerned land masses (*d_i_*_,*j*_). As *d* → ∞, everything becomes disconnected. If *d* = 0, everything is well mixed, regardless of landmass distinctions;
— the colonization parameter *c* > 0 enables us to control the extent of dispersal. As *c* → 0, everything becomes disconnected, and the basic model without dispersal is recovered. As *c* → ∞, everything is well mixed, regardless of distance;
— the values of *C_i_*_,*j*_ in the special case when *i* *=* *j* are uniquely defined by recognizing that the entries in each row of the matrix represent the probabilities of all possible outcomes of a random process, so must sum to unity.

### (e) Continental fragmentation and drift

Terrestrial tetrapods appear in the fossil record shortly before the formation of Pangaea [33], making Pangaea the optimal starting point for our model. The current geological eon, the Phanerozoic, has seen drastic changes in land mass configuration with near constant shifting of the geological landscape over time by means of mantle convection [34]. We simulated the break up of Pangaea in the mid-Jurassic followed by the movement of the resulting continents according to an explicit scheme. In brief, the initial rift in Pangaea, which eventually formed the North Atlantic Ocean, was followed by the splitting of Gondwana into Gondwana East and West, which subsequently broke apart into seven continents: Australia, Africa, Eurasia, North America, South America, Antarctica and India [2,3]. These continents later fused to form today's land masses; see figure 1 and electronic supplementary material, appendix 2 for further details.

Distances between pairs of continents were approximated through the use of Google maps (www.google.co.uk/maps/) for the present day and based upon the literature [3,35–38] for historic time points. Distances were measured between the coasts of continental mainlands, ignoring island chains such as the archipelagos of South East Asia. The distance between continents depended continuously on time because of the gradual process of continental drift. In the absence of detailed information (which in any case would be over-elaborate for the simplified approach taken here), we assumed a constant speed of continental movement implemented as a linear interpolation between adjacent time points when distances could be estimated. We assume that individuals could not cross an intermediate land mass in a single dispersal event. However, a series of consecutive dispersal events could together enable a lineage to hop across multiple continents.

Sea-level fluctuations can result in land mass areas changing over time. This is extensive for areas of land such as the Indo-Australian archipelago [39]. However, accurately predicting land area over deep time and global scales would be difficult and beyond the scope of this manuscript given, for example, the uncertainty of potential erosion of the exposed strata [40]. The current land-mass areas were therefore assumed to be uniform throughout the simulation (full tables of intercontinental distances and areas are shown in electronic supplementary material, appendix 2).

Our simulations of the geologically explicit version of the model were conducted for a range of community sizes *J*_M_, speciation rates *v* and dispersal parameters *c* to test for robustness to these choices. We focused some simulations particularly on the small values of *v* intended to indicate the behaviour of higher taxa.

### (f) Vicariance speciation depending on distance

We also ran more advanced simulations in which further speciation may be caused by vicariance in addition to the background rate of speciation given by *υ*. This vicariance speciation results from the colonization of a land mass different from an individual's starting location and should depend on the distance travelled [41]. We chose probability of speciation by vicariance *f*, upon any intercontinental dispersal event over a distance *d*, to be given by
2.8
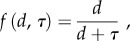
where *τ* is a parameter indicating a distance-dependent inhibition to vicariance speciation. When *τ* = 0, all dispersal events result in vicariance speciation, whereas as *τ* → ∞ vicariance speciation never occurs, regardless of the value of *d*. For intermediate values of *τ*, increasing *d* increases the probability of vicariance speciation, as *d* → ∞, vicariance speciation always occurs, regardless of the value of *τ*. This scenario considered the possible effects of isolation between continents in driving vicariance and thereby speciation. We use the closest distances between two unconnected land masses in the current day as our value for *τ*. We therefore run simulations where *τ* = 1000, where 1000 km is the distance between South America and Antarctica. For our simulation code written in R, see the electronic supplementary material.

## 3. Results

**Figure 2.**
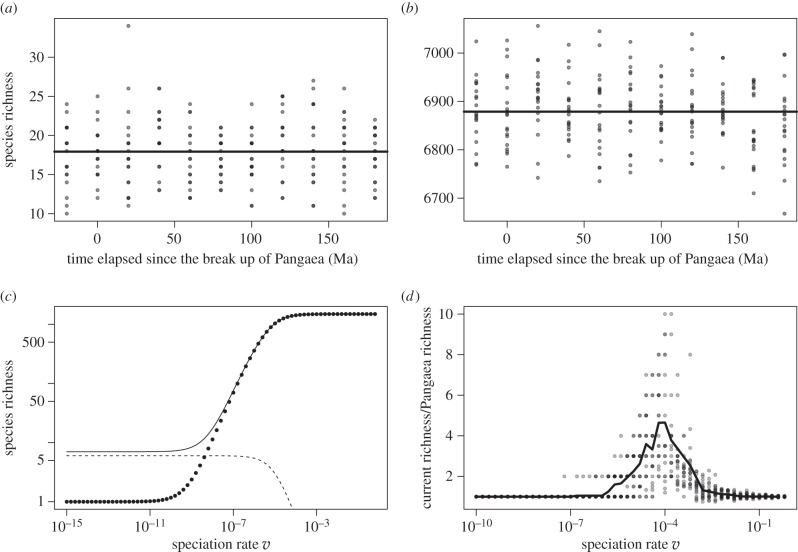
Evaluation of the model with no dispersal between continents. (*a,b*) Simulations of the species richness in the system as a function of time for *v* = 0.00001 and *g* = 1; (*a*) results for *δ* = 0.001, sampling = 1 and (*b*) *δ* = 1, sampling = 0.001. (*c*) By analytical evaluation of equation (2.3), the species richness (solid line for sum of continents assuming infinite time since break of Pangaea, dots for Pangaea), and gain in species richness from continental fragmentation (dashed line) as a function of *v*, for *g* = 1, *δ* = 10 and sampling = 0.00001. (*d*) Simulations of the proportion of species richness gain resulting from continental drift expected at the present day 180 Myr after the breakup of Pangaea as a function of speciation rate *v* (for density *δ* = 0.0001; *g* = 1 and sampling = 1).

Simulations for the simplest model in which continents are completely isolated showed a constant species richness over time from the break up of Pangaea to the present day (figure 2*a*). This constant trend is robust to different parametrizations of density and sampling (figure 2*b*). Evaluation of equation (2.3) shows the same pattern consistently for almost all parameter choices: the gain in diversity from the splitting of Pangaea remains relatively constant at 6 and only decreases for very large and unrealistic values of *v*. When total diversity is small, the relative increase brought about by continental fragmentation is more notable (figure 2*c*). Simulations show three regimes upon changing *v*: (i) when *v* is large, richness is high and not significantly affected by continental drift; (ii) when *v* is smaller richness is lower and is significantly increased by continental drift; and (iii) when *v* is sufficiently small the system is only represented by a single species, regardless of continental drift (figure 2*d*).

Assuming *z* = 0.8, equation (2.6) based on SARs yields a ratio of current to Pangaea species richness of 1.42. If *z* = 0.2, then the ratio is increased to 4.53. However, the data in figure 1 give a ratio in excess of 6 indicating that SARs are not a sufficient explanation in isolation, even when *z* is increased to unrealistically high values for continental scales.

**Figure 3.**
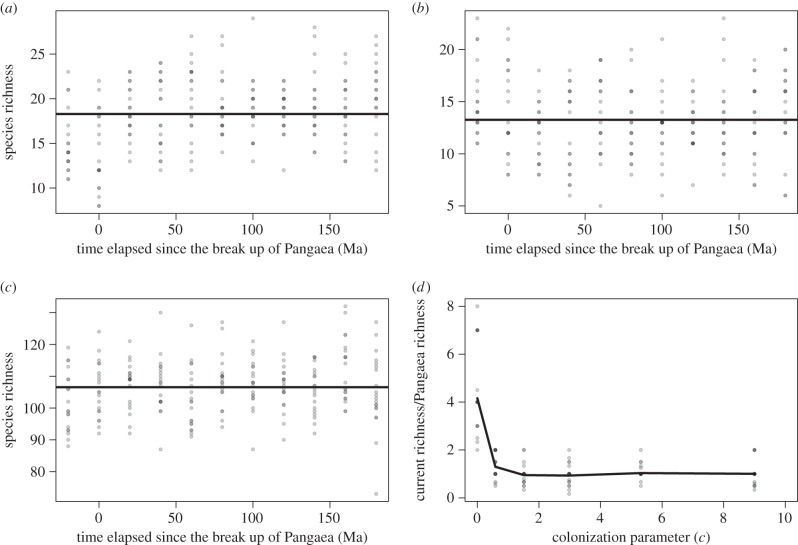
Simulations of the model with dispersal between continents for a geologically explicit scenario. (*a*–*c*) Species richness over time for *g* = 1, *δ* = 0.0001 and sampling = 1. The individual panels show different values of speciation rate *v* and dispersal parameter *m* as follows: (*a*) *v* = 0.0001, *c* = 1; (*b*) *ν* = 0.0001, *c* = 1000; (*c*) *v* = 0.001, *c* = 1000. (*d*) The proportion of species richness gain resulting from continental drift as a function of *c* (for *v* = 0.0001, *δ* = 0.00001 and sampling = 1).

The model incorporating dispersal between continents along with an explicit and geologically accurate history of continental drift does not change the observation that species richness is typically constant over time (figure 3*a–c*). This observation also remains robust to changes in density *δ*, speciation rate *v* and the colonization parameter *c*. In the special case where *v* becomes small enough for total diversity to be of the same order as the number of continents, continental drift does have a notable effect on diversity that can be eroded by increasing the colonization parameter (*c*; figure 3*d*).

**Figure 4.**
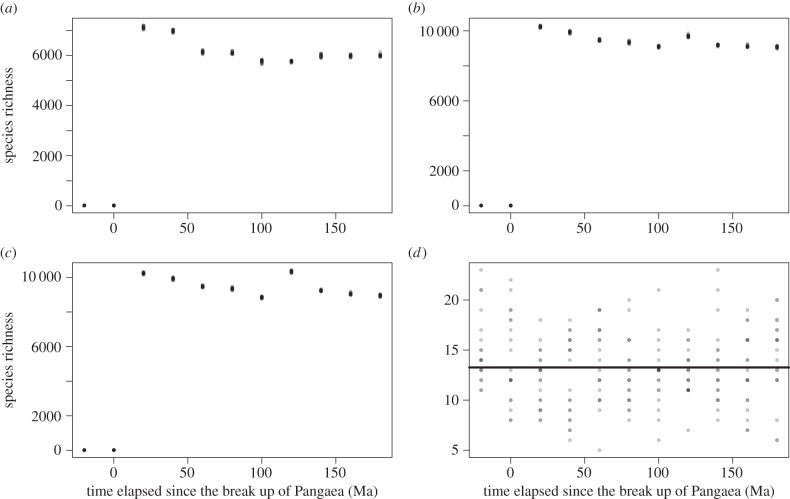
Simulations as for figure 3, but with dispersal between continents invoking speciation (*δ* = 0.0001, sampling = 1, *v* = 0.0001). (*a*) *g* = 1, *c* = 1 and *τ* = 0; (*b*) *g* = 1, *c* = 1000 and *τ* = 0; (*c*) *g* = 50, *c* = 1000 and *τ* = 0; (*d*) *g* = 1, *c* = 1000 and *τ* = 1000.

Incorporating vicariance speciation as well as background speciation can show increasing richness over time to a new equilibrium level as the distance between continents increases (figure 4). The panmictic nature of Pangaea in our model means that no vicariance-mediated speciation occurred prior to its fragmentation. As such, any increase in richness post the break up of Pangaea is as a result of dispersal influenced vicariance speciation. Shorter generation lengths can produce an overshoot in diversity through time, which after an initial peak decreases to its equilibrium level. The longer the generation length, the slower the increase caused by dispersal (figure 4*b,c* and electronic supplementary material, appendix 3). Generation length does not affect the final equilibrium richness of the system, but it does change the timescale within which it will be reached and furthermore damps the system preventing the possibility of a peak and then a decline in diversity. In the more general case where *τ* > 0, so that intercontinental dispersal only results in speciation with a probability increasing with distance, the extent to which vicariance increases richness is reduced. When *τ* = 1000, we see the effects of vicariance speciation completely negated (figure 4*d*).

## 4. Discussion

We have investigated whether continental fragmentation could cause the increase in species richness observed in the fossil record based purely on neutral processes of dispersal, speciation and drift among ecologically equivalent species. Our simplest neutral model considered the effects of the breakup of Pangaea into completely isolated continents with no dispersal between them. An analytical approximation for this model showed the diversity expected prior to the break up of Pangaea was normally not notably different to that achieved at the new equilibrium after the break up of Pangaea. The same was true of a SAR model for large values of the parameter *z* that are expected at such scales (e.g. *z* = 0.8). Even with smaller values of *z*, the SAR relationship could not produce a sufficient increase in diversity post break up to match the empirical data.

When total diversity in the neutral model became small (similar to the number of continents), a significant increase in diversity is observed as a result of continental drift. This is because in the extreme case of low speciation rate, after a sufficiently long period of time, a fragmented set of continents necessarily maintains a separate species in each, whereas a single supercontinent would only contain one species. As the speciation rate decreases still further, the system ultimately converges to a state where there is still only one global species, because the speciation event bringing it into existence is pushed back to a time when the continents were still connected. While these scenarios are not realistic for species, they could be appropriate for higher taxa. Moreover, despite the fact that named higher taxa are relatively arbitrary taxonomic units, they are the units used for most studies of the fossil record, meaning that the increase in richness observed is mirrored to some extent in the fossil record. In mammals, supra-orders are associated with different continental regions (e.g. *Afrotheria*, *Xenarthra*, etc.), albeit at a far coarser level of taxonomic resolution than is attainable in fossil studies. Similarly, continental fragmentation will lead to higher phylogenetic diversity than in a single continental area. The date of the most recent common ancestor of all species in a single area is set by the model, whereas the divergence between clades isolated in different continents is expected to increase linearly with the time passed since their isolation [42].

Dispersal alone was not enough to change the predictions based on isolated continents apart from in special cases where global diversity was low (figure 3). In such cases, isolation of continents gave rise to endemicity that could not be sustained in a well-mixed system of the same total size. Increasing dispersal in such a system reduces the scope for species to coexist as endemics alongside competing species from other continents that have the dispersal ability to invade. Our results were generally extremely robust to changes in parameters; this goes some way to justifying the application of neutral theory because it suggests that a system with multiple different guilds each with their own parameters would still produce the same results, both for individual guilds and for the sum behaviour of a large number of such guilds together.

For large metacommunity size and relatively high speciation rate, the only scenario leading to a change in species richness as Pangaea split was when speciation is associated with migration. This effectively represents lineages colonizing novel environments, and speciating in allopatry. In order to obtain an increase in species richness since the break up of Pangaea, high values of both dispersal and speciation were required in the model. When the generation length was relatively long, relaxation of the system to a higher global diversity became a more gradual process as the biological system moves more slowly. When generation length was shorter, so that the biological system moves much faster than the underlying geological changes, species richness was observed to peak and then decrease again to a new equilibrium over time. This ‘overshoot’ pattern of diversity through time has been observed in spiders on the Hawaiian islands by performing space for time substitutions [43]. It has also been recorded in simulation models of diversification [44]. In our model, the observed overshoot is caused by the fact that after the continents split apart, species richness shoots up because of the relatively fast pace of the biological system and the increased net diversification rates. After this, continents drifting further apart result in less dispersal between them and hence fewer opportunities for speciation and declining equilibrium species richness.

There is significant potential for further work extending these models. The spatial structure of regions within continents could be taken into account thus relaxing the rather strong assumption that individuals are well mixed within continents. Such a model would have the effect of increasing *N* in equations (2.1)–(2.4), because *N* would correspond to the number of regions rather than the number of continents. This in turn may cause the flat relationship between richness and time to break down as *N* becomes comparable to global species richness if it refers to (possibly small) regions within continents rather than whole continents. Our work could also be further developed to consider shallow-marine taxa in particular which have indeed experienced an increase in habitable area as a result of continental drift.

## 5. Conclusion

We conclude that continental fragmentation is unlikely to explain the observed increase in terrestrial species richness purely through neutral processes of dispersal and isolation. There are alternative, non-neutral effects of fragmentation that might explain changes in diversity. For example, tectonic changes such as the separation of Antarctica from South America led to the establishment of the Benguela current and led to progressive cooling and drying of world climates (figure 1). These climate changes might have led to environmental conditions that favoured increased speciation and/or decreased extinction of tetrapods, for example by creating new temperate habitat types that could be easily colonized [45]. Present-day spatial patterns of diversity associate warm and wet habitats with higher diversity for mammals, birds and amphibians [46] (reptiles tend to be more diverse in warm and dry habitats). In contrast, global climate has cooled and dried recently during the biggest spike in tetrapod diversity when if anything diversity should decrease. Local climates might still have changed, however, in ways that make them more favourable for diversification. For example, the interiors of continents have become less arid since the break up of Pangaea because of closer access to moist oceanic air. Increased area of coastal regions might also have opened up new habitat areas. The split of Pangaea is well documented in affecting clade dispersal and isolation of clades. In principle, these might influence patterns of diversity. However, with a basic neutral model, we see that it has no effect on the species richness of the system. This holds true for nearly all sizes of global community and values of *v*. The theoretical exception is when *v* is so small that each continent tends to contain a single unit of diversity; this is not realistic for species but potentially relevant for higher taxa and phylogenetic diversity. The incorporation of dispersal alongside generation length yields no change in richness. If, however, dispersal leads to an increased probability of speciation, an increase in species richness over time is possible. The values required for this to occur though are improbable. Thus, we postulate that continental drift by itself is not sufficient to account for the increase in terrestrial biodiversity observed in the fossil record whether acting through neutral mechanisms or through classic SAR.

## Supplementary Material

Appendices 1–3

## Supplementary Material

Appendix 4
