# Supplementary material for: Quantifying the effects of the break up of Pangaea on global terrestrial diversification with neutral theory
Source: Philos Trans R Soc Lond B Biol Sci. 2016 Apr 5;371(1691):20150221. doi: 10.1098/rstb.2015.0221 (PMC4810815; doi:10.1098/rstb.2015.0221)
Supplement: Appendices 1–3 [file rstb20150221supp1.pdf]

Supplementary material for Jordan, Sean and Barraclough, Tim and Rosindell, James, 2016, Quantifying the effects of the break up of Pangaea on global terrestrial diversification with neutral theory, *Phil. Trans. R. Soc. B.* doi: 10.1098/rstb.2015.0221.

Appendix 1 : Further details on the analytical solution

Consider Pangaea containing J_M individuals and splitting into N smaller landmasses. Take J_i for $1 \leq i \leq N$ to indicate the number of individuals in each of the smaller landmass, assume these sum to the same total as Pangaea $\sum_{i=1}^{N} J_i = J_\mathrm{M}$, so the total number of individuals in the system is constant. We wish to calculate the global gain in diversity from this split, assuming all the newly split continents reach their own equilibrium richness. Equation 4 from the main text gives us a near approximation of species richness S in Pangaea:

$$S \approx 1 + J_\mathrm{M}\nu \cdot ln\left(1 + \frac{\mathrm{x}}{\upsilon}\right) \qquad \text{(Equation 4)}$$

Recall that x is the probability of sampling an individual and ν is the per-capita speciation rate. Similarly we can calculate S_i for $1 \leq i \leq N$ which gives the diversity in each of the smaller landmasses

$$S_i \approx 1 + J_\mathrm{i}\nu \cdot ln\left(1 + \frac{\mathrm{x}}{\upsilon}\right) \qquad \text{(Equation A1)}$$

The total gain in diversity G is given by

$$G = \left(\sum_{i=1}^{N} S_i\right) - S \qquad \text{(Equation A2)}$$

Now insert Equations 4 and A1 into Equation A2

$$G = \left(\sum_{i=1}^{N}\left(1 + J_\mathrm{i}\nu \cdot ln\left(1 + \frac{\mathrm{x}}{\upsilon}\right)\right)\right) - \left(1 + J_\mathrm{M}\nu \cdot ln\left(1 + \frac{\mathrm{x}}{\upsilon}\right)\right)$$

Multiply out the brackets and remove the 1 to be outside the sum

$$G = N + \left(\sum_{i=1}^{N}\left(J_\mathrm{i}\nu \cdot ln\left(1 + \frac{\mathrm{x}}{\upsilon}\right)\right)\right) - 1 - J_\mathrm{M}\nu \cdot ln\left(1 + \frac{\mathrm{x}}{\upsilon}\right)$$

Remove the other multiples from the sum

$$G = N + \left(\sum_{i=1}^{N}(J_\mathrm{i})\right) \cdot \nu \cdot ln\left(1 + \frac{\mathrm{x}}{\upsilon}\right) - 1 - J_\mathrm{M}\nu \cdot ln\left(1 + \frac{\mathrm{x}}{\upsilon}\right)$$

Replace $\left(\sum_{\mathrm{i}=1}^{\mathrm{N}}(\mathrm{J_i})\right)$ with $\mathrm{J_M}$

$$G = N + J_\mathrm{M}\nu \cdot ln\left(1 + \frac{\mathrm{x}}{\upsilon}\right) - 1 - J_\mathrm{M}\nu \cdot ln\left(1 + \frac{\mathrm{x}}{\upsilon}\right)$$

Cancel terms

$$G = N - 1 \qquad \text{(Equation A3)}$$

This is the result quoted in the main text.

© The Authors under the terms of the Creative Commons Attribution License http://creativecommons.org/licenses/by/3.0/, which permits unrestricted use, provided the original author and source are credited.

Supplementary material for Jordan, Sean and Barraclough, Tim and Rosindell, James, 2016, Quantifying the effects of the break up of Pangaea on global terrestrial diversification with neutral theory, *Phil. Trans. R. Soc. B.* doi: 10.1098/rstb RSTB-2015-0221.R1.

Appendix 2: Geographical and geological details

Geology
The current geological Eon, the Phanerozoic, has seen drastic changes in land mass configuration. It began c.545-540 Ma with the Cambrian [1-3], a time of massive diversification in metazoans [1,3,4]. This is further divided into Eras representative of major stages in the macroscopic fossil record, typified by mass extinction events [5,6], with the most famous being the Mesozoic Era and its dinosaurs. During the Phanerozoic, there was a near constant shifting of the geological landscape over time by means of mantle convection [7]. This process gave rise to various supercontinents over time such as Pangaea and Rodinia. Pangaea was the first super continent that terrestrial Tetrapods came across [8] and is therefore the starting point of our model.
A brief overview, as shown in figure 1 maps A-H of the main manuscript, is as follows:

A. 180 Ma - N. America rifts from Africa forming the N. Atlantic Ocean
B. 165 Ma - Gondwana East (Antarctica, Australia, India) splits from Gondwana West (Africa, S. America)
C. 135 Ma - S. America rifts apart from Africa.
D. 132 Ma – India breaks off from Antarctica and Australia
E. 80 Ma – Australia breaks off from Antarctica
F. 55 Ma – India collides wit Asia in the Himalayan orogenic event. (An orogenic event is a mountain forming event.)
G. 33 Ma – Drake Passage formed as S. America separates from Antarctica
H. 12 Ma – Isthmus of Panama formed

The landmasses studied are (complete with land area)

- Africa, 30,221,532 Km^2
- Antarctica, 14,000,000 Km^2
- Australia, 7,692,024 Km^2
- Eurasia, 51,471,000 Km^2
- India, 3,288,000 Km^2
- North America 24,709,000 Km^2
- South America, 17,840,000 Km^2

For reference, this then gives the following in sum:

- Gondwana (East – Antarctica, Australia and India. West – Africa and S. America) 73,041,556 Km^2
- Laurasia (Eurasia and N. America) 76,180,000 Km^2
- Pangaea 149,221,556 Km^2

© The Authors under the terms of the Creative Commons Attribution License http://creativecommons.org/licenses/by/3.0/, which permits unrestricted use, provided the original author and source are credited.

Supplementary material for Jordan, Sean and Barraclough, Tim and Rosindell, James, 2016, Quantifying the effects of the break up of Pangaea on global terrestrial diversification with neutral theory, *Phil. Trans. R. Soc. B.* doi: 10.1098/rstb RSTB-2015-0221.R1.

CURRENT	Africa	Antarctica	Australia	Eurasia	India	N. America	S. America
Africa	0	3853	7615	0	4711	4538	2840
Antarctica	3853	0	3005	6280	8081	9370	1000
Australia	7615	3005	0	1350	4700	11180	8950
Eurasia	0	6280	1350	0	0	82	5459
India	4711	8081	4700	0	0	18700	21800
N. America	4538	9370	11180	82	18700	0	0
S. America	2840	1000	8950	5459	21800	0	0

Table A1: Current day distances (Km) from one continent to another. as used in our simulation code (Appendix 3). These are the shortest direct routes between continents without crossing other continents.

PANGAEA	Africa	Antarctica	Australia	Eurasia	India	N. America	S. America
Africa	0	0	1700	0	0	0	0
Antarctica	0	0	0	11500	0	13000	0
Australia	1700	0	0	3500	0	24000	6000
Eurasia	0	11500	3500	0	3400	0	14500
India	0	0	0	3400	0	28000	29000
N. America	0	13000	24000	0	28000	0	0
S. America	0	0	6000	14500	29000	0	0

Table A2: Estimated distances (Km) from one another once Pangaea has begun rifting at 180 Ma and is no longer considered panmictic.

© The Authors under the terms of the Creative Commons Attribution License http://creativecommons.org/licenses/by/3.0/, which permits unrestricted use, provided the original author and source are credited.

Supplementary material for Jordan, Sean and Barraclough, Tim and Rosindell, James, 2016, Quantifying the effects of the break up of Pangaea on global terrestrial diversification with neutral theory, *Phil. Trans. R. Soc. B.* doi: 10.1098/rstb RSTB-2015-0221.R1.

The following two tables are examples of the dispersal matrix used in the simulations. The starting location for the randomly chosen individual is show on the left (and indexed as numbers in the Appendix 3 code - hence the numbers for labels). The dispersal probabilities are then calculated for each row based upon the current point in time. From this the current intercontinental distances are determined and so are the probabilities. The sum of the 6 dispersal probabilities for end location other than the individual's starting one is then subtracted from 1 to produce the probability of an individual's movement being intracontinental as opposed to resulting in a colonisation.

	Oz	Af	EA	NA	SA	An	In
(Australia) 1	9.997e-01	2.659e-05	2.553e-04	1.481e-05	1.336e-05	3.121e-05	4.687e-06
(Africa) 2	6.768e-06	6.550e-01	3.449e-01	3.648e-05	4.208e-05	2.434e-05	4.676e-06
(Eurasia) 3	3.816e-05	2.025e-01	7.734e-01	1.995e-03	2.186e-05	1.493e-05	2.203e-02
(N. America) 4	4.610e-06	4.462e-05	4.156e-03	8.762e-01	1.196e-01	1.001e-05	1.178e-06
(S. America) 5	5.759e-06	7.129e-05	6.317e-05	1.656e-01	8.342e-01	9.372e-05	1.010e-06
(Antarctica) 6	1.715e-05	5.255e-05	5.492e-05	1.767e-05	1.194e-04	9.997e-01	2.726e-06
(India) 7	1.097e-05	4.298e-05	3.449e-01	8.854e-06	5.484e-06	1.161e-05	6.550e-01

Table A3: Dispersal matrix at delta = 0.001, sampling = 0.001 for *c* = 1 and 100 Ma after the breakup of Pangaea.

	Oz	Af	EA	NA	SA	An	In
1	0.860	0.011	0.010	0.001	0.002	0.094	2.203e-02
2	0.003	0.370	0.345	0.047	0.120	0.094	2.203e-02
3	0.001	0.203	0.635	0.158	0.001	0.001	6.826e-04
4	0.000	0.058	0.330	0.492	0.120	0.001	8.055e-05
5	0.001	0.203	0.002	0.166	0.535	0.094	7.572e-05
6	0.052	0.203	0.003	0.001	0.120	0.600	2.203e-02
7	0.052	0.203	0.011	0.203	0.000	0.094	6.403e-01

Table A4: Dispersal matrix at delta = 0.001, sampling = 0.001 for *c* = 100 and 10 Ma after the breakup of Pangaea.

© The Authors under the terms of the Creative Commons Attribution License http://creativecommons.org/licenses/by/3.0/, which permits unrestricted use, provided the original author and source are credited.

Supplementary material for Jordan, Sean and Barraclough, Tim and Rosindell, James, 2016, Quantifying the effects of the break up of Pangaea on global terrestrial diversification with neutral theory, *Phil. Trans. R. Soc. B.* doi: 10.1098/rstb RSTB-2015-0221.R1.

References

1. Signor, Philip W (1992) Origin and early evolution of the Metazoa Springer Science, 10,

2. Grotzinger, John P and Bowring, Samuel A and Saylor, Beverly Z and Kaufman, Alan J (1995) Biostratigraphic and geochronologic constraints on early animal evolution, Springer Science, 598–598,

3. Knoll, Andrew H and Walter, Malcolm R (1992) Latest Proterozoic stratigraphy and Earth history

4. Dalziel, Ian WD (1997) OVERVIEW: Neoproterozoic-Paleozoic geography and tectonics: Review, hypothesis, environmental speculation, Geol Soc of Am, 109(1):16–42.

5. Stanley, SM and Yang, Xiangling (1994) A double mass extinction at the end of the Paleozoic era, Science, 266(5189):1340–1344,

6. Bramlette, MN (1965) A double mass extinction at the end of the

Paleozoic era, Science, 148(3678):1696–1699,1965

7. Gurnis, Michael (1988) Large-scale mantle convection and the aggregation and dispersal of supercontinents, Nature, 332(6166):695–699.

8. Romer, Alfred S (1958) Tetrapod limbs and early tetrapod life, Evolution, 12 (3): 365–369, 22.

© The Authors under the terms of the Creative Commons Attribution License http://creativecommons.org/licenses/by/3.0/, which permits unrestricted use, provided the original author and source are credited.

Appendix 3: Additional simulation results

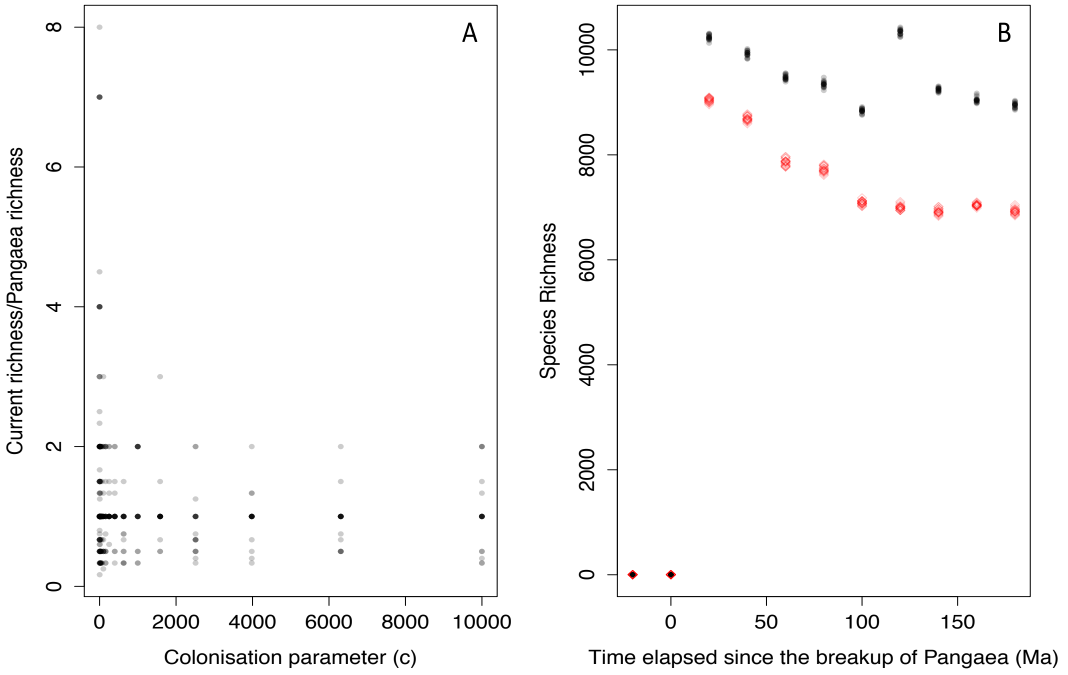

Figure A1: Simulations with geologically explicit information. Panel A shows the full simulation of more broadly varying *c* values as described in figure 3D of the main text; this demonstrates the proportion of species richness gain resulting from continental drift as a function of *c* (for ν = 0.0001, δ = *0.00001* and sampling = *1*). Panel B shows simulations as for Figure 4 (main text), but with δ = *0.0001*, sampling = 1, ν = 0.00001. Both sets of simulations (red and black points) consist of a community with a generation length of 300, but *c* = 1000 for those in black and *c* = 100 for those in red.

© The Authors under the terms of the Creative Commons Attribution License http://creativecommons.org/licenses/by/3.0/, which permits unrestricted use, provided the original author and source are credited.

Supplementary material for Jordan, Sean and Barraclough, Tim and Rosindell, James, 2016, Quantifying the effects of the break up of Pangaea on global terrestrial diversification with neutral theory, *Phil. Trans. R. Soc. B.* doi: 10.1098/rstb RSTB-2015-0221.R1.

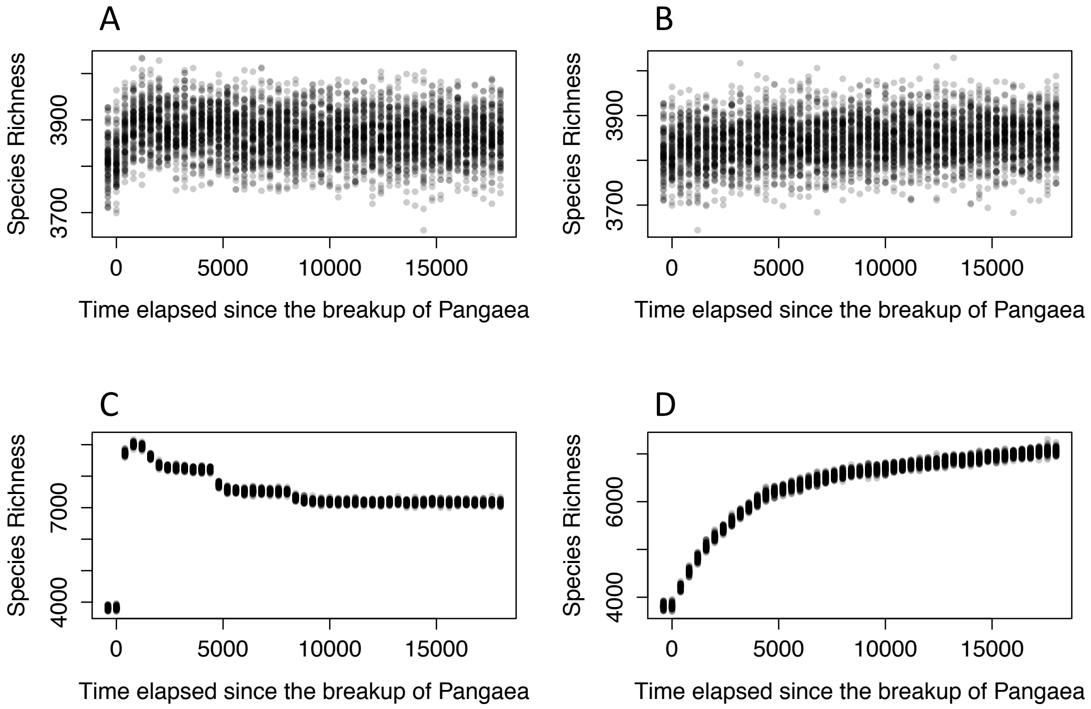

Figure A2: Showing species richness through time as for Figure A1 above and Figure 4 of the main text. These were run for much shorter time scales (as shown in the x axis) and with speciation rate ν = 0.1. Panels A and C show g =1 whilst B and D show g = 33.3. Panels A and B show c = 0.01 whilst C and D show c = 1.

© The Authors under the terms of the Creative Commons Attribution License http://creativecommons.org/licenses/by/3.0/, which permits unrestricted use, provided the original author and source are credited.
